# Mitral apparatus assessment by delayed enhancement CMR - relative impact of papillary muscle and left ventricular wall infarction on ischemic mitral regurgitation

**DOI:** 10.1186/1532-429X-14-S1-P100

**Published:** 2012-02-01

**Authors:** Parag Goyal, Jason Chinitz, Fahmida Islam, Debbie W Chen, Sean Wilson, Prashanth Venkatesh, Matthew D Cham, Thanh Nguyen, Yi Wang, Richard B Devereux, Jonathan W Weinsaft

**Affiliations:** 1 Cardiology, Weill Cornell Medical College, New York, NY, USA; 2 Radiology, Mount Sinai Medical Center, New York, NY, USA; 3 Radiology, Weill Cornell Medical College, New York, NY, USA

## Summary

Lateral wall infarction on DE-CMR, independent of papillary muscle involvement, confers increased risk for ischemic mitral regurgitation.

## Background

The mitral apparatus contains two myocardial components - papillary muscles and the adjacent left ventricular (LV) wall. Delayed enhancement CMR (DE-CMR) enables in-vivo study of potential contributions of LV wall and papillary muscle infarction (PMI) to MR. This study examined the relative impact of papillary muscle and LV wall infarction on mitral regurgitation (MR) following ST elevation MI (STEMI).

## Methods

Multimodality imaging was prospectively performed among patients with first STEMI: DE-CMR (IR-GRE, acquired 10-30 minutes post gadolinium [0.2 mmol/kg]) was used to assess LV infarct pattern - including PMI (graded by location and extent - complete or partial, stratified using threshold of >50% papillary myocardium) and LV wall infarction (17 segment model, 5 point score/segment). Cine-CMR (SSFP) was analyzed for cardiac function and geometry - including LV chamber size, regional wall motion (5 point score/segment), and mitral annular diameter. Echocardiography (echo) was used to quantify MR (0-4+ grade) using established consensus criteria. Each imaging modality was read independently.

## Results

**Figure 1:**
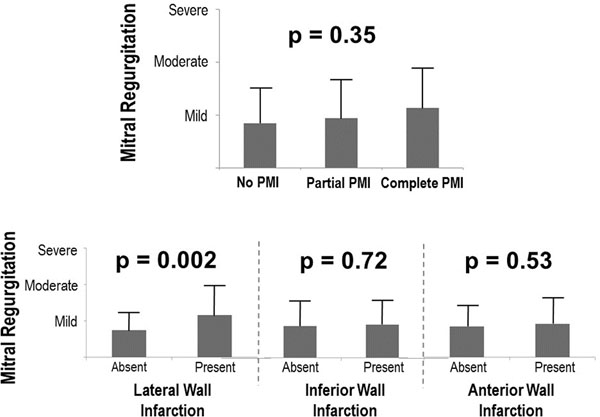
Mitral regurgitation severity stratified by papillary muscle infarction (top) and LV wall infarct distribution (bottom).

153 patients (57±12yo, 81% male) with first STEMI were studied. Echo and CMR (1.5T) were performed within 1 day in all patients (27±8 days post STEMI). 42% had MR (mean grade: 0.9±0.7, 14% with ≥ 2+). MR severity was similar among patients with and without PMI (p=0.35; Figure 1, top), and did not differ by PMI location (p=0.93). MR varied by LV infarct distribution, with severity greatest in patients with lateral wall infarction on DE-CMR (p=0.002), but non-significant differences when patients were stratified based on inferior (p=0.72) or anterior (p=0.53) wall involvement (Figure 1, bottom). Lateral wall infarct size was over three-fold larger (12.8±4.2% LV myocardium) among patients with bilateral PMI compared to those with isolated posteromedial (4.4±4.4%), anterolateral (1.3±3.0%), or absent (0.8±2.0%) PMI (p<0.001), paralleling a trend towards increased MR severity (p=0.054) in the bilateral PMI group. In multivariable analysis (Table 1), MR (≥ 2+) was independently associated with lateral wall infarct size (OR 1.29/% LV myocardium, p=0.004), mitral annular diameter (OR 10.23/cm, p=0.009) and LV chamber diameter (OR 3.06/cm, p=0.049), with a trend towards inverse association for PMI (OR 0.34, p=0.08). Substitution of lateral wall motion score (OR 1.27, p=0.005) in the model also demonstrated an association between lateral wall dysfunction and MR, as well as a non-significant association for PMI (OR 0.51, p=0.18).

**Table 1:** Structural correlates of mitral regurgitation.

Variable	Odds ratio	95% confidence interval	P
Lateral wall infarct size (% myocardium)	1.29	1.09-1.53	**0.004**
Papillary muscle infarction	0.34	0.10-1.12	0.08
LV end-diastolic diameter (cm)	3.06	1.01-9.29	**0.049**
Mitral annular diameter (cm)	10.23	1.78-58.76	**0.009**

Model *χ2* = 30.99, p<0.001.

## Conclusions

Lateral wall injury - whether assessed by infarct size or contractile dysfunction - confers increased risk for MR following STEMI, even after controlling for both mitral annular and LV chamber geometry. Neither presence nor location of PMI independently impacts severity of post-STEMI MR.

## Funding

K23 HL102249-01, Lantheus Medical Imaging, Doris Duke Clinical Scientist Development Award.

